# Association and Risk Factors for Obstructive Sleep Apnea and Cardiovascular Diseases: A Systematic Review

**DOI:** 10.3390/diseases9040088

**Published:** 2021-12-02

**Authors:** Amal K. Mitra, Azad R. Bhuiyan, Elizabeth A. Jones

**Affiliations:** Department of Epidemiology and Biostatistics, School of Public Health, College of Health Sciences, Jackson State University, Jackson, MS 39213, USA; azad.r.bhuiyan@jsums.edu (A.R.B.); elizabeth.jones@students.jsums.edu (E.A.J.)

**Keywords:** sleep apnea, cardiovascular disease, hypertension, diabetes, age, obesity, risk factors, apnea-hypoxia index (AHI)

## Abstract

Obstructive sleep apnea (OSA) is a serious, potentially life-threatening condition. Epidemiologic studies show that sleep apnea increases cardiovascular diseases risk factors including hypertension, obesity, and diabetes mellitus. OSA is also responsible for serious illnesses such as congestive heart failure, stroke, arrhythmias, and bronchial asthma. The aim of this systematic review is to evaluate evidence for the association between OSA and cardiovascular disease morbidities and identify risk factors for the conditions. In a review of 34 studies conducted in 28 countries with a sample of 37,599 people, several comorbidities were identified in patients with severe OSA—these were: heart disease, stroke, kidney disease, asthma, COPD, acute heart failure, chronic heart failure, hyperlipidemia, thyroid disease, cerebral infarct or embolism, myocardial infarction, and psychological comorbidities including stress and depression. Important risk factors contributing to OSA included: age > 35 years; BMI ≥ 25 kg/m^2^; alcoholism; higher Epworth sleepiness scale (ESS); mean apnea duration; oxygen desaturation index (ODI); and nocturnal oxygen desaturation (NOD). Severe OSA (AHI ≥ 30) was significantly associated with excessive daytime sleepiness and oxygen desaturation index. The risk of OSA and associated disease morbidities can be reduced by controlling overweight/obesity, alcoholism, smoking, hypertension, diabetes mellitus, and hyperlipidemia.

## 1. Introduction

Sleep disordered breathing, alternatively called obstructive sleep apnea (OSA), is one of the most important biological cause of chronic sleep fragmentation and sleep deficiency [1]. OSA syndrome represents a challenge for the healthcare system due to its high prevalence in the adult population [2,3]. It causes intermittent nocturnal hypoxemia, excessive daytime sleepiness, and persistent elevated sympathetic tone during the day [1]. In a population-based cluster sampling of Sao Paulo inhabitants in Brazil [3], the prevalence rate of OSA was 32.8% (95% confidence intervals (CI), 29.6% to 36.3%), one of the highest rates observed compared to other epidemiologic studies. OSA rates were higher in men than women (odds ratio (OR) = 4.1; 95% CI, 2.9 to 5.8; *p* < 0.001) and in obese individuals (OR = 10.5; 95% CI, 7.1 to 15.7; *p* < 0.001) compared with individuals of normal weight [3]. The prevalence studies indicate that sleep disturbances including chronic sleep deprivation and sleep fragmentation due to various environmental and biological factors lead to mood disorders, anxiety disorders, poor cognition, memory deficiency, and decreased performance in academia and the workplace [4,5,6].

OSA is a significant risk factor for major cardiovascular diseases, including arterial hypertension, ischemic heart disease, heart failure, rhythm/conduction disturbances, as well as cerebral stroke [7,8]. The underlying mechanisms explaining associations between OSA and cardiovascular disease are not entirely delineated. Important risk factors for OSA include obesity, craniofacial or oropharyngeal anatomic abnormalities, male sex, and smoking [8]. During sleep, there is a reduction in tone of the dilator muscles involved in maintaining airway patency. Obesity that results in relative narrowing of the airway lumen increases the likelihood of obstruction [8]. Several intermediary pathways might be involved: these include sustained sympathetic activation, intrathoracic pressure changes, and oxidative stress [9]. Among other mechanisms, the role of disorders in coagulation factors, endothelial damage, platelet activation, and increased inflammatory mediators are considered in the pathogenesis of cardiovascular diseases [9,10].

Severe OSA is an independent predictor for risk for all-cause and cardiovascular mortality irrespective of race and ethnicity [11]. However, according to a recent Jackson Heart study, African Americans had a higher prevalence of hypertension and resistant or uncontrolled hypertension, both of which may be partially explained by untreated OSA [12]. The National Commission on Sleep Disorders Research estimated that sleep apnea is probably responsible for 38,000 cardiovascular deaths yearly, and approximately over USD 42 million is spent on hospitalizations due to cardiovascular diseases [13]. Among the cardiovascular diseases, OSA increases the risk of heart failure by 140%, the risk of stroke by 60%, and the risk of coronary heart disease by 30% [14].

Among the biological factors, several studies have documented a higher prevalence and complication of OSA in African American populations [15,16,17]. However, in-depth studies to examine the cardiovascular risk and associated comorbidities due to OSA in the general population are scanty. The primary objective of this systematic review is to elucidate the associated risk factors for OSA and cardiovascular diseases, with particular attention to hypertension, diabetes, and obesity. In this review, we will also assess the relationship of OSA with other disease comorbidities as potential risk factors for cardiovascular diseases.

## 2. Materials and Methods

The study addressed recently published peer reviewed original studies (but no review articles) on cardiovascular diseases, primarily focusing on hypertension and its associated comorbidities and obstructive sleep apnea. The inclusion and exclusion of the review are mentioned in Table 1.

### 2.1. Search Guidelines

The primary search engines that were used to select articles included PubMed, EBSCOhost, Scopus, Cochrane Library, Google Scholar, MEDLINE, Alt HealthWatch, CINAHL Consumer Health Complete, and Health Source: Nursing/Academic Edition with full text. The studies were selected for the review based on inclusion criteria such as (1) English only, (2) human studies, (3) scholarly papers, (4) age groups 19 and older, (5) had cardiovascular diseases with or without comorbidities, and (6) had sleep apnea. The search was performed in April–September 2021. The time limit for the studies included in this review was from 2018–2021. The search terms used included: ‘sleep apnea’ OR ‘obstructive sleep apnea’ or ‘apnea’ OR ‘hypertension’ OR ‘high blood pressure’ OR ‘cardiovascular diseases’ OR ‘risk factors’ OR ‘comorbidities’.

### 2.2. Screening Guidelines

**Figure 1:**
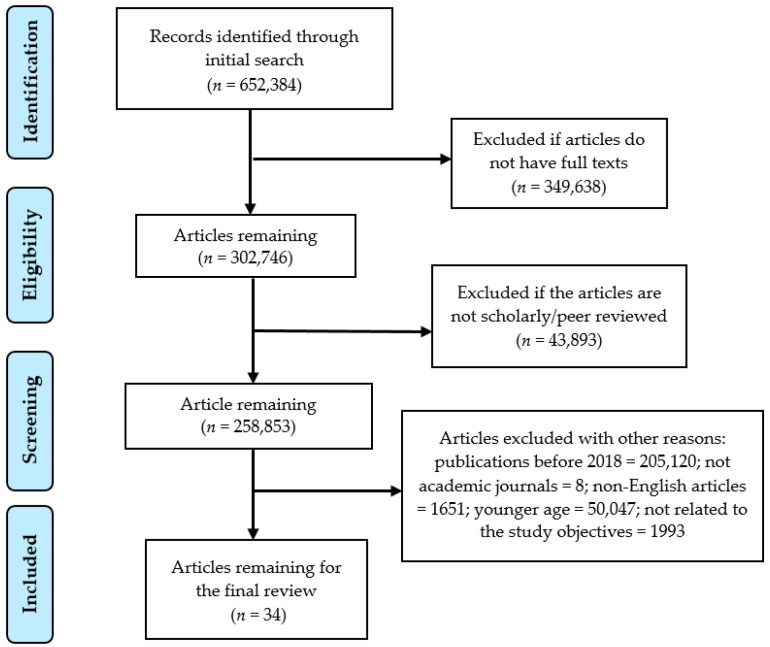
PRISMA flow chart to illustrate the article search and the inclusion process.

The Preferred Reporting Items for Systematic Reviews and Meta-Analysis (PRISMA) guidelines (2009) [18] was followed to document the review process. Selected abstracts were assessed to make sure that the studies met the eligibility criteria for inclusion. Three researchers (AKM, ARB, and EAJ) independently screened the titles and retrieved the abstracts based on the inclusion criteria and exclusion criteria as mentioned in Table 1. Selected abstracts were reviewed to assess their eligibility for a full text review. Studies were included in the review if a consensus was achieved by all three researchers. Conflicts were controlled by having group discussions between all three of the researchers of this study. During the second phase of screening, articles without a full text and review articles were excluded. After discussion, more articles were excluded because they were not available in full text, were not inclusive of an adult population (19+), and were not directly related to hypertension and sleep apnea. Finally, all the researchers agreed upon 34 studies that were identified for further evaluation. The PRISMA flow chart (Figure 1) exhibits the search and the inclusion and the exclusion process for the systematic review.

### 2.3. Quality Appraisal

Studies were appraised for quality by all three of the researchers separately based on a pre-determined rating scale. The rating scale from 0 to 4 was based on the following criteria: (1) study design: cross-sectional, case–control, or cohort study = 1, otherwise = 0; (2) sample size: large = 1, small = 0; (3) selection of samples: random selection or lack of bias = 1, non-random or convenience sample or presence of bias = 0; and (4) analyses: robust (such as multivariate analyses) = 1, otherwise = 0. We followed the similar rating scales published in an earlier study [19]. Based on the above-mentioned criteria, the three researchers rated each of the 34 articles independently from a range of 0 to 4 and categorized as low (a score of 0–1), moderate (a score of 2–3), or high (a score of 4). An average value of the three scores was presented as the final score, because there were no significant inter-observer variations in the assessment of the quality of the included articles (Table 2).

**Table 2:** Study design, diseases addressed, major findings, and the quality appraisal of the articles.

Author [Ref]	Country of Study	Study Design	Sample Size	Cardiovascular and Other Comorbidities Addressed	Major Findings	Quality Appraisal (0 to 4)
Almeneessier et al., 2020 [20]	Saudi Arabia	Cross-sectional study	32; mean age of 50.2 ± 12 years	Hypertension	Mean Apnea-Hypoxia Index (AHI) was 40.1 ± 27.6 events/h. AHIs were 38.3 ± 30.6 and 51.9 ± 28.3 events/h for non-rapid eye movement (NREM) and rapid-eye movement (REM) sleep, respectively. No differences were detected in obstructive respiratory event duration or degree of desaturation between REM and NREM sleep. Oxygen desaturation index was a predictor of increased systolic blood pressure during obstructive events and post-obstructive event period.	Score = 2; moderate; study design = 1 robust analyses = 1; others = 0.
Alvarez-Sabín et al., 2017 [21]	Spain	Matched cross-sectional study	183	Hypertension with or without silent cerebral infarct; obesity	The frequency of severe OSA syndrome (AHI > 30) was 44.3% in patients with silent cerebral infarct (SCI) and 38.5% in the control group. Severe OSA syndrome is highly prevalent among hypertensive subjects and is independently associated with lacunar silent cerebral infarct.	Score = 2; moderate; study design = 1 robust analyses = 1; others = 0.
Barreto et al., 2020 [22]	Brazil	Prospective cohort study	102 (male = 64%)	Ischemic stroke; hypertension; diabetes; heart disease; physical inactivity	Wake-up stroke occurred in 1 of 3 of cases. Irrespective of stroke, half of the patients had moderate to severe sleep apnea. Type 2 diabetes mellitus was independently associated with stroke (OR = 2.76; CI: 1.10–6.05; *p* = 0.03).	Score = 2; moderate; study design = 1 robust analyses = 1; others = 0.
Borsini et al., 2018 [23]	Argentina	Prospective follow-up study for one year	168	Hypertension; obesity with a mean BMI of 34.7 ± 6.79; and diabetes mellitus	Of the individuals with hypertension, 44% had an AHI of >15 events/h, and CPAP was recommended in 69 (41%) patients. Based on the American College of Cardiology/American Heart Association (ACC/AHA) Risk Calculator, there was a gradual increase in the risk of cardiovascular events (coronary event or stroke) proportional to the increase in AHI severity categories: 8.7% for patients with normal AHI; and 19.7%, 27.8%, and 30.3% for patients with OSA of 5.1–14.9, 15–29.9, and ≥30, respectively.	Score = 2; moderate; study design = 1 robust analyses = 1; others = 0.
Choudhury et al., 2019 [24]	India	Community based cross-sectional study	200	Hypertension, obesity	Patients with high risk of OSA, snoring was reported by 70%, excessive daytime sleepiness by 50%, 28% were smokers and 48% reported alcohol use. The associated risk factors were age > 35 years (OR = 4.5, 95% CI, 1.4 to 13.8), BMI ≥ 25 kg/m^2^ (aOR = 3.5, 95% CI, 1.2 to 10.5), alcoholism (aOR = 4.5, 95% CI, 1.8 to 11.1), and hypertension (aOR = 11.5, 95% CI, 4.7 to 28.0).	Score = 3; moderate; study design = 1, sample selection = 1, robust analyses = 1; sample size = 0.
Cohen et al., 2018 [25]	United States	Prospective follow-up study	3101	Narcolepsy, hypertension, thyroid disease, obesity, hyperlipidemia, anxiety, peripheral neuropathy, diabetes mellitus	There was an increased association of narcolepsy with anxiety, thyroid disease, hypertension, and hyperlipidemia. At the end of 9.9 years (SD 7.27 years), there was increased association of narcolepsy with peripheral neuropathy, glucose intolerance, and automobile-related trauma. OSA symptoms were significantly increased in the narcolepsy group as compared to controls (at diagnosis: OR = 69.25, 95% CI = 9.26 to 517.99, *p* < 0.0001; at end (after about 10 years) OR = 13.55, 95% CI = 5.08 to 36.14, *p* < 0.0001).	Score = 4; high; study design, sample size, sample selection and data analyses, each scored 1.
Coussa-Koniski et al., 2020 [26]	Lebanon	Prospective longitudinal study	663	Hypertension; coronary artery disease; myocardial infarction; stroke; arrhythmias; chronic heart failure; asthma; COPD; diabetes mellitus; kidney disease; depression; overweight/obesity.	Comorbidities were reported by 90% of the patients with 60% reporting 2–4 comorbidities. The severity of OSA was higher in men compared with women; however, comorbidities were higher in women. In multiple regression, increasing age (OR = 1.91, 95% CI, 1.22–3.0), male gender (OR = 3.16, 95% CI, 1.86–5.39), and obesity (OR = 2.39, 95% CI, 1.53–3.73) associated with hypertension and diabetes, symptoms of snoring, excessive daytime somnolence, and witnessed apneas were significantly associated with severe sleep apnea. Significant declines in systolic and diastolic blood pressure, intima-media thickness, and renal resistance index and increase in the left ventricular ejection fraction were observed after a 6-month therapy with CPAP.	Score = 3; moderate; study design = 1, sample size = 1, robust analyses = 1; sample selection = 0.
Frigy et al., 2019 [27]	Romania	Cross-sectional study (hospital based)	51 consecutive cases (13 women and 38 men) (mean age 60.8 years).	Acute heart failure; diabetes mellitus; obesity; renal dysfunction.	Patients underwent 24-h combined monitoring of ECG and blood pressure and sleep apnea testing before discharge. The presence of significant sleep apnea was found to influence blood pressure and nocturnal ventricular ectopy in patients with stabilized acute heart failure.	Score = 2; moderate; study design = 1, sample selection = 1; others = 0.
Ghesquière et al. [28]	United States	Prospective longitudinal study	67 pregnant women	Obesity (mean BMI of 42.4 ± 6.2 kg/m^2^); hypertension.	29 pregnant women (43%) had OSA (95% CI, 31.4 to 55.2%); mild or moderate OSA was in 25 (37%) women and severe OSA in 4 (6%). Women in the OSA group were older, had chronic hypertension more frequently, and had a higher mean BMI.	Score = 2; moderate; study design = 1, robust analyses = 1; others = 0.
Gürün et al., 2020 [29]	Turkey	Cross-sectional study	266; normal blood pressure (*n* = 125) and high blood pressure (*n* = 141).	Hypertension	Hypertension is associated OSA. The risk factors included: older age (OR: 1.095, 95% CI 1.05 to 1.14), higher Epworth sleepiness scale (ESS) (OR: 1.19, 95% CI 1.07 to 1.31), mean apnea duration (OR: 1.07, 95% CI 1.03 to 1.11), oxygen desaturation index (ODI) (OR: 1.06, 95% CI 1.03 to 1.10), and nocturnal oxygen desaturation (NOD) (OR: 2.44, 95% CI 1.17 to 5.09).	Score = 2; moderate; study design = 1, robust analyses = 1; others = 0.
Haarmann et al., 2019 [30]	Germany	Prospective longitudinal study (10 years)	378	Hypertension, diabetes mellitus, atherosclerotic disease, and chronic heart failure; 206 (60%) had OSA.	OSA was classified as none (AHI < 5/h), mild (AHI 5–14/h), moderate (AHI 15–29/h), or severe (AHI ≥ 30/h). Overall mortality was higher in the OSA group (14.9% vs. 5.9%; *p* = 0.007); however, the mortality difference disappeared after adjustment for age and sex. The study did not find evidence of an adverse effect of OSA on cardiovascular morbidity and mortality.	Score = 4; high; study design, sample size, sample selection and data analyses, each scored 1.
Hein et al., 2019 [31]	Belgium	Hospital-based cross-sectional study	703	Major depression; hypertension	Multivariate logistic regression analysis revealed that severe objective insomnia, low complaints of repeated nighttime awakenings or early morning awakening, and intermediate or low self-reported insomnia complaints were significant risk factors of high blood pressure in major depression.	Score = 4; high; study design, sample size, sample selection and data analyses, each scored 1.
Hein et al., 2019 [32]	Belgium	Cross-sectional study	1272	Hypertension	The prevalence of high blood pressure with insomnia was 30.03%. Short sleep duration (<5 h), severely reduced sleep efficiency (<65%), high sleep fragmentation (sleep fragmentation index ≥ 18/h), and long-term use of short or intermediate half-life benzodiazepine receptor agonists were significant risk factors for hypertension in individuals with insomnia.	Score = 3; moderate; study design = 1, sample size = 1, robust analyses = 1; sample selection = 0.
Hobzova et al., 2018 [33]	Czech Republic	Cohort study; multicenter sample selection	188	Hypertension; obesity	AHI was classified as mild for AHI = 5–14.9, moderate for AHI = 15–29.9 and severe for AHI ≥ 30. Undiagnosed sleep apnea (SA) was found in 72.9% patients (29.3% mild, 26.6% moderate, 17.0% severe) patients with blood pressure. Moderate/severe SA (AHI ≥ 15) was associated with BMI ≥ 30 kg/m^2^ (OR = 6.22, 95% CI, 3.10–12.49), and central obesity (OR = 3.73, 95% CI, 1.57–8.83), but not with ambulatory blood pressure.	Score = 3; moderate; study design = 1 sample selection = 1; robust analyses = 1; others = 0.
Hsu H.-C. et al., 2018 [34]	Taiwan	Cross-sectional study (hospital based)	215	Hypertension	Nearly 82% of the hypertensive participants were found having undiagnosed OSA, 80% of which were of mild to moderate severity. Severe OSA was significantly associated with excessive daytime sleepiness (OR, 20.27; 95% CI, 1.58 to 26.97) and oxygen desaturation index (OR, 4.05; 95% CI, 1.86 to 8.81).	Score = 2; moderate; study design = 1 robust analyses = 1; others = 0.
Johnson et al., 2019 [12]	United States	Cross-sectional study	913 African Americans (mean age of 64.0 ± 10.6 years).	Hypertension; obesity (58.6%); diabetes mellitus (28.2%).	Overall, 48% of participants had uncontrolled hypertension and 14% had resistant hypertension. Untreated African American patients with moderate or severe sleep apnea had an increased risk of resistant hypertension. Severe OSA (AHI ≥ 30) compared with no OSA (AHI < 5) was associated with a 3.5 times higher odds of resistant hypertension (95% CI, 1.54 to 7.91). After adjustment for confounders, individuals with moderate or severe sleep apnea had a 2.0 times higher odds of resistant hypertension (95% CI, 1.14 to 3.67).	Score = 4; high; study design, sample size, sample selection and data analyses, each scored 1.
Karhu et al., 2021 [35]	Finland	Prospective longitudinal study	2535 Sleep Heart Health Study participants	Hypertension (*n* = 1164), diabetes (*n* = 170) and cardiovascular diseases (*n* = 265)	The increase in oxygen desaturation index (β = 2.73, 95% CI: 1.15 to 4.32, *p* = 0.001) and desaturation duration (β = 1.85, 95% CI: 0.62 to 3.08, *p* = 0.003) were higher among participants with cardiovascular diseases. These results suggest that patients with pre-existing diabetes or cardiovascular diseases are at increased risk for an expedited worsening of intermittent hypoxemia.	Score = 4; high; study design, sample size, sample selection and data analyses, each scored 1.
Kim et al., 2018 [36]	Korea	Prospective cohort study	1825	Hypertension	The prevalence of hypertension was higher among subjects with OSA and with high homocysteine (Hcy) levels. After adjusting for confounding factors, subjects with OSA and high Hcy levels had a 1.86-fold risk of developing hypertension compared to those without OSA and high Hcy levels.	Score = 4; high; study design, sample size, sample selection and data analyses, each scored 1.
Kolluri et al., 2020 [37]	United States	Retrospective study	264	Chronic venous disease (CVD), diabetes mellitus, pulmonary embolism, renal insufficiency	22.7% of the patients had elevated central venous pressure (CVP) and 26.9% had OSA. There was no significant difference in the prevalence of OSA. The predictors of elevated CVP were age > 64.6 years (OR, 1.03; 95% CI, 1.003 to 1.05; *p* = 0.026), diabetes mellitus (OR, 2.19; 95% CI, 1.05–4.5; *p* = 0.035), and right lower extremity Venous Clinical Severity Score of ≥8.5 (OR, 1.098; 95% CI, 1.011–1.193; *p* = 0.026).	Score = 3; moderate; study design = 1, sample size = 1, robust analyses = 1; sample selection = 0.
Lai et al., 2019 [38]	Italy	Prospective longitudinal study	24	Resistant hypertension; overweight	Elderly patients (mean age of 60.3 ± 9.9 years, mean BMI of 28.7 ± 3.3 kg/m^2^) with resistant hypertension and OSA were controlled through 6-month long treatment with continuous positive airway pressure (CPAP). The treatment was considered effective if the patient was compliant, and the AHI was reduced by ≥50% from baseline or below 10 events per hour.	Score = 2; moderate; study design = 1 robust analyses = 1; others = 0.
Li et al., 2018 [39]	China	Cross-sectional study	144	Hypertension	There were no significant differences between subjects with and without morning hypertension in sleep disturbances and sleep respiratory parameters.	Score = 2; moderate; study design = 1 robust analyses = 1; others = 0.
Louis et al., 2018 [40]	United States	Cross-sectional study	3264 women in early pregnancy and 2512 women in late pregnancy	Hypertension, obesity (*n* = 380, 11.8%), morbid obesity (*n* = 349, 10.8%) hypothyroidism (*n* = 172, 5.5%)	The prevalence of sleep-disordered breathing (SDB) was 3.6% and 8.3% in early pregnancy and late pregnancy, respectively. Logistic regression models showed age, BMI, and frequent snoring as predictors of SDB.	Score = 4; high; study design, sample size, sample selection and data analyses, each scored 1.
Manov et al., 2018 [41]	Bulgaria	Cross-sectional study	279; 162 (58%) men; mean age 42.8 ± 12.4 years	Pericardial effusion in patients without PH; obesity (mean BMI of 37.3 ± 7.8 kg/m^2^).	Severity of OSA was graded as follows: mild (AHI 5–15), moderate (AHI 15–30), and severe (AHI > 30). Pericardial effusion was found in 102 (36.56%)—all of them with moderate to severe OSA syndrome. A significant positive correlation was found between the presence of pericardial effusion and AHI (r = 0.374, *p* < 0.001), BMI (r = 0.473, *p* < 0.001), and desaturation time during sleep (r = 0.289, *p* < 0.001).	Score = 4; high; study design, sample size, sample selection and data analyses, each scored 1.
Morinaga et al., 2018 [42]	Japan	Cross-sectional study	2208 men (mean age, 44.4 ± 0.2 years)	Hypertension	The prevalence of mild-to-moderate (AHI of 5–29) and severe (AHI ≥ 30) OSA in the studied subjects were 7.1%, and 6.1%, respectively. Associations between hypertension and OSA were observed after adjustments for age, body mass index (BMI), estimated glomerular filtration rate, current alcohol intake, current smoking habits, and OSA treatment.	Score = 4; high; study design, sample size, sample selection and data analyses, each scored 1.
Nozato et al., 2019 [43]	Japan	Cross-sectional study	29 patients (aged 65 years or older)	Hypertension; obesity.	The patients were categorized into two groups: no-to-mild OSA and moderate-to-severe OSA, by using the respiratory disturbance index (RDI) and 3% oxygen desaturation index (ODI). BMI was correlated with RDI and 3% ODI (*r* = 0.56 and 0.43, respectively). Sleep apnea severity was associated with pulse rate variability but not with blood pressure variability.	Score = 2; moderate; study design = 1 robust analyses = 1; others = 0.
Paine et al., 2019 [44]	New Zealand	Cross-sectional study	12,500 adults	Hypertension, obesity, diabetes mellitus, heart disease	The prevalence of each sleep complaint measure was highest for the indigenous Māori population. Reporting any sleep complaint was associated with higher odds of poorer mental health, diagnosed high blood pressure, diagnosed diabetes, diagnosed heart disease, poor/fair self-rated health, obesity, current smoking, and hazardous drinking.	Score = 3; moderate; study design = 1, sample size = 1, robust analyses = 1; sample selection = 0.
Poka-Mayap et al., 2020 [45]	Cameroon	Cross-sectional hospital based study	359 (56% women)	Hypertension, diabetes mellitus, acute heart failure, stroke, HIV infection, and epilepsy.	The prevalence of high risk of OSA and hypopnea syndrome (OSAHS), as assessed from the STOP-BANG questionnaire, was 64.1% (95% CI, 59.1 to 69.1%) in the whole population, 78.3% (95% CI, 71.9 to 81.7%) in men and 53% (95% CI, 46.1 to 59.9%) in women (*p* < 0.001). Moderate to severe sleep apnea was associated with hypertension (OR, 3.24; 95% CI, 1.08 to 9.72; *p* = 0.036.	Score = 4; high; study design, sample size, sample selection and data analyses, each scored 1.
Saraei et al., 2020 [46]	Iran	Cross-sectional study	281 locomotive drivers (mean age of 43 ± 10 years)	Hypertension, obesity	Mean BMI was 26.9 ± 3.9 kg/m^2^; 166 (59.9%) drivers had two or more of the following risk factors: blood pressure ≥ 140/90 mmHg, history of drug use, BMI > 35 kg/m^2^, age > 50 years, and neck circumference > 40 cm.	Score = 1; low; study design = 1; others = 0.
Schiavone et al., 2019 [47]	Argentina	Cross-sectional clinic-based study	382 patients; 234 men (61.3%) and 148 women; Mean age: 54.5 ± 13.7 years.	Hypertension; obesity (BMI, 33.1 ± 7.8 kg/m^2^)	Adult patients were recruited from a hypertension center. Among the patients analyzed, 57.2% had moderate to severe OSA, while 42.8% had mild OSA. The high-risk Berlin questionnaire was the most sensitive tool to identify AHI > 5 eV/hour, while STOP-BANG questionnaire (SBQ) > 5 performed better at identifying patients with >15 eV/hour with a high discrimination power.	Score = 4; high; study design, sample size, sample selection and data analyses, each scored 1.
Seguro et al., 2018 [48]	France	Retrospective case–control study	200; normal blood pressure (*n* = 100), high blood pressure (*n* = 100).	Hypertension; obesity (*n* = 126, 63%); asthma (*n* = 5, 3%), and COPD (*n* = 8, 4%).	Cases were hypertensive patients and controls were age- and gender-matched normotensive patients. Severe OSA was defined as an AHI ≥ 30. OSA was considered symptomatic when ESS > 10. Associations were found between resistant hypertension and untreated obstructed sleep apnea. However, individuals with hypertension were less symptomatic with episodes of sleep apnea with a lower rate of the ESS (10.0 vs. 11.9, *p* < 0.01).	Score = 2; moderate; study design = 1 robust analyses = 1; others = 0.
Sun et al., 2019 [49]	China	Cross-sectional study	106	COPD; pulmonary hypertension (PH) mean age 69.5 years, 91.5% men	56 (52.8%) patients with COPD were diagnosed with OSA, and 24 (22.6%) patients with COPD had PH. Multiple regression analysis showed a significant and independent effect of both FEV_1_% predicted and AHI on PH, indicating declining lung function and increased severity of OSA in patients with COPD.	Score = 2; moderate; study design = 1 robust analyses = 1; others = 0.
Sunwoo et al., 2018 [50]	Korea	Cross-sectional study	2740	Hypertension, diabetes, hyperlipidemia, myocardial infarction, angina pectoris, other heart diseases, stroke and psychiatric comorbidities.	The prevalence of a “high risk” for OSA was 15.8% (95% CI, 14.5 to 17.2%). Multiple logistic regression analysis showed that BMI ≥ 25 kg/m^2^ (OR 10.75, 95% CI, 8.21–14.06), hypertension (OR = 5.83, 95% CI, 3.91–8.69), diabetes (OR = 2.54, 95% CI, 1.46–4.42), hyperlipidemia (OR = 2.85, 95% CI, 1.36–5.95), and anxiety (OR = 1.63, 95% CI, 1.03–2.59) were significantly related with a “high risk” for OSA, whereas physical activity ≥ 3 times/week (OR = 0.85, 95% CI, 0.66–1.09) had a protective effect.	Score = 4; high; study design, sample size, sample selection and data analyses, each scored 1.
Wächter et al., 2020 [51]	11 Sleep centers in 8 European countries	Multicenter retrospective cohort study	1877 (OSA patients and healthy controls)	Hypertension; obesity	Effects of OSA severity and potential confounders on sleep architecture were small, but transition patterns still linked sleep fragmentation directly to OSA-related clinical outcomes such as arterial hypertension and daytime sleepiness.	Score = 4; high; study design, sample size, sample selection and data analyses, each scored 1.
Wilson et al., 2018 [52]	Australia	Cross-sectional study	80 women (40 cases and 40 controls)	Gestational hypertension, pre-eclampsia	The frequency of sleep-disordered breathing was 1.4 times higher in the cases than controls (52.5% vs. 37.5%, respectively; *p* = 0.18). Severity of sleep-disordered breathing was more than twice as common in women with gestational hypertension or pre-eclampsia compared to controls (35% vs. 15%, *p* = 0.039).	Score = 2; moderate; study design = 1 robust analyses = 1; others = 0.

## 3. Results

A summary of the study design, the countries of the studies, major findings regarding the correlation between cardiovascular disease with sleep apnea, comorbidities, and the quality appraisal of the studies are presented in Table 2. The 34 studies included in the systematic review comprised of 37,599 participants. In this review, the studies represented 28 countries including Argentina, Australia, Belgium, Brazil, Bulgaria, Cameroon, China, Czech Republic, Finland, France, Germany, Greece, India, Iran, Italy, Ireland, Japan, Korea, Lebanon, New Zealand, Poland, Romania, Spain, Saudi Arabia, Slovakia, Taiwan, Turkey, and the United States. A multicenter study, conducted by Wächter et al., 2020 [51], represented 11 sleep centers in 8 European countries.

### 3.1. Sample Size

The sample sizes varied widely, mainly due to the differences in the study methodologies. However, a few notable studies involving large sample sizes were as follows: a cross-sectional study with 12,500 adults conducted by Paine et al. in New Zealand in 2019 [44]. In this study, the authors showed the highest prevalence of sleep disturbances among the indigenous Māori population. The associated comorbidities for sleep apnea included poorer mental health, high blood pressure, diabetes mellitus, heart disease, obesity, current smoking, and hazardous drinking. The second largest cross-sectional study (*n* = 5776 women) was conducted by Louis et al. in the United States [39], in which increasing age, obesity, and snoring were identified as predictors of sleep-disordered breathing.

Two other large studies, both being of prospective cohort design, included 3101 in a study in the United States [25] and 2535 in a Finnish study [35]. In the US study [25], OSA symptoms were significantly increased in patients with narcolepsy as compared to controls at diagnosis (OR = 69.25, 9.26 to 517.99, *p* < 0.0001) at nearly 10 years of follow-up (OR = 13.55, 5.08 to 36.14, *p* < 0.0001). The Finnish study [35] identified a significant association of pre-existing diabetes and cardiovascular diseases with an increased risk for an expedited worsening of intermittent hypoxemia. Another cross-sectional study involving 2740 adults in Korea [50] identified the “high risk” individuals as being older aged and having a BMI ≥ 25 kg/m^2^ for OSA. Several other studies that involved large samples included a cross-sectional study (*n* = 2208) in Japan addressing the association between hypertension and OSA [42], a multicenter study (*n* = 1877) in Europe looking for the effects of OSA severity and other comorbidities on sleep pattern [51], a prospective cohort study (*n* = 1825) in Korea determining the association between hypertension with OSA in patients with high levels of homocysteine in the blood [36], and a cross-sectional study (*n* = 1272) in Belgium determining the prevalence of hypertension with insomnia [32].

### 3.2. Study Design

Of the 34 studies, the majority were cross-sectional studies (*n* = 21, 62%), 10 (29%) were prospective cohort studies, and 3 (9%) were retrospective studies. Of the cross-sectional studies, 5 were performed in a hospital or a clinic setting, and 16 were community-based studies. Only one study by Seguro et al., in France [48] proposed a case–control design by comparing OSA severity measured by AHI scores among 100 cases with hypertension and 100 age- and gender-matched normotensive individuals as controls. In that study, an association was found between resistant hypertension and untreated obstructed sleep apnea.

### 3.3. Major Study Findings

#### 3.3.1. Risk Factor for OSA

Studies consistently reported several risk factors contributing to OSA. These include: age > 35 years (OR = 4.5, 95% CI, 1.4 to 13.8) [23]; BMI ≥ 25 kg/m^2^ (aOR = 3.5, 95% CI, 1.2 to 10.5) [24]; alcoholism (aOR = 4.5, 95% CI, 1.8 to 11.1) [24]; higher Epworth sleepiness scale (ESS) (OR: 1.19, 95% CI 1.07 to 1.31) [29]; mean apnea duration (OR: 1.07, 95% CI 1.03 to 1.11) [29]; oxygen desaturation index (ODI) (OR: 1.06, 95% CI 1.03 to 1.10) [29]; and nocturnal oxygen desaturation (NOD) (OR: 2.44, 95% CI 1.17 to 5.09) [29]. Severe OSA (AHI ≥ 30) was significantly associated with excessive daytime sleepiness (OR, 20.27; 95% CI, 1.58 to 26.97) and oxygen desaturation index (OR, 4.05; 95% CI, 1.86 to 8.81) [34].

Based on this review, several modifiable risk factors for OSA were identified. These include: overweight/obesity, alcoholism, smoking, hypertension, diabetes mellitus, and hyperlipidemia.

#### 3.3.2. Bidirectional Association between OSA and Hypertension

The prevalence of hypertension was higher among subjects with OSA and with high homocysteine (Hcy) levels. After adjusting for confounding factors, subjects with OSA and high Hcy levels had a 1.86-fold risk of developing hypertension compared to those without OSA and high Hcy levels [36]. On the other hand, severe OSA syndrome was highly prevalent among hypertensive subjects [21]. Of the individuals with hypertension, 44% had an AHI of >15 events/h, and CPAP was recommended in 69 (41%) patients [23]. However, no causal association could be established based on the nature of the studies.

#### 3.3.3. AHI Severity and Cardiovascular Risk

Based on the ACC/AHA risk calculator, there was a gradual increase in the risk of cardiovascular events (coronary event or stroke) proportional to the increase in AHI severity categories: 8.7% for patients with normal AHI; and 19.7%, 27.8%, and 30.3% for patients with OSA of 5.1–14.9, 15–29.9, and ≥30, respectively [23]. The French study by Seguro et al. [48] is the only case–control study reviewed here. It is noteworthy that the authors reported a lower Epworth sleepiness scale (ESS) in hypertensive patients than in normotensive patients with severe OSA, which could be misleading. The authors, therefore, suggested estimating the OSA severity by polysomnography, even if the ESS score is low in patients with hypertension [48].

#### 3.3.4. OSA and Comorbidities

**Figure 2:**
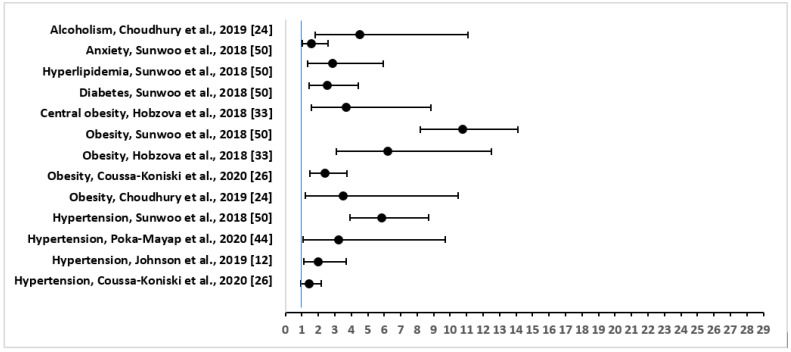
Forest Plot of Odds Ratio with 95% Confidence Intervals for Risk Factors or Comorbidities of Severe Obstructive Sleep Apnea.

The three most common comorbidities that were reported were: hypertension in 30 out of 34 (88%), followed by overweight/obesity in 17 (50%), and diabetes mellitus in 12 (35%) of the studies. Other important physical conditions that were associated with OSA included heart disease (six studies), stroke (three studies), kidney disease including renal insufficiency (three studies), asthma (two studies), COPD (two studies), acute heart failure (two studies), chronic heart failure (two studies), hyperlipidemia (two studies), thyroid disease (two studies), cerebral infarct or embolism (two studies), myocardial infarction (two studies), and psychological comorbidities including stress, anxiety, and depression (three studies). Figure 2 shows the adjusted OR and 95% CI of the comorbidities or risk factors for severe OSA.

Among other comorbidities, in a study in Spain [21], severe OSA syndrome was highly prevalent among hypertensive subjects, irrespective of having silent cerebral infarct. In a prospective longitudinal study in Lebabon [26], out of 663 patients, the comorbidities of OSA included: coronary artery disease (*n* = 65); myocardial infarction (*n* = 6); arrhythmias (*n* = 66); chronic heart failure (*n* = 34); asthma (*n* = 52); COPD (*n* = 69); kidney disease (*n* = 36); and depression (*n* = 69) [26]. Patients with sleep apnea were also associated with pericardial effusion (36.6%) in a cross-sectional study in Bulgaria [41]. A significant positive correlation was found between the presence of pericardial effusion and AHI (*r* = 0.374, *p* < 0.001), BMI (*r* = 0.473, *p* < 0.001), and desaturation time during sleep (*r* = 0.289, *p* < 0.001) [41]. In a prospective follow-up study of patients with OSA in the US [25], there was increased association of narcolepsy with peripheral neuropathy, glucose intolerance, and automobile-related trauma. OSA symptoms were significantly increased in the narcolepsy group as compared to controls (at diagnosis: OR = 69.25, 95% CI = 9.26 to 517.99, *p* < 0.0001; at the end of about 10 years of follow-up: OR = 13.55, 95% CI = 5.08 to 36.14, *p* < 0.0001) [25].

### 3.4. Quality Appraisal

The mean ± SD score for the quality of the studies was 2.82 ± 1.06. Of the 34 studies, the majority (*n* = 20, 59%) scored moderate, 13 (39%) scored high, and one (3%) scored low [46]. Although the Iranian study [46] scored low, the study provided data on cardiovascular risk factors of OSA in a special population (locomotive drivers), which are pertinent to this review.

## 4. Discussion

This systematic review showed that the severity of OSA is directly associated with major risk factors for cardiovascular disease, including hypertension, overweight and obesity, diabetes mellitus, hyperlipidemia, and physical inactivity. The review also identified several risk factors for OSA, such as increasing age, BMI ≥ 25 kg/m^2^, alcoholism, smoking, higher ESS score, mean apnea duration, oxygen desaturation index, and nocturnal oxygen desaturation. Severe OSA was significantly associated with excessive daytime sleepiness and oxygen desaturation index.

Heart disease is the leading cause of death worldwide. Therefore, assessing the risk of its occurrence is a crucial step in predicting serious cardiac events. The characteristics of cardiovascular disease are identified by its primary risk factors. Those factors are diabetes mellitus, the level of lipids in the blood, coronary artery function, and kidney function [53]. A data mining model was developed with a clinical laboratory database using a naïve Bayes classifier to detect cardiovascular risk, and it was tested for its accuracy in predicting three levels of risk [53]. Our review results are aligned with the sophisticated data mining model as proposed in determining the risk of cardiovascular disease.

Women’s risk assessment for cardiovascular disease is equally important. Traditional atherosclerotic cardiovascular disease (ASCVD) risk factors, such as hypertension, diabetes mellitus, hypercholesterolemia, and obesity, affect both sexes, but some may affect women differently and are considered to be more potent [54]. Although our primary focus is to identify the relationship between OSA and cardiovascular disease, our review described an Australian study that demonstrated the association of gestational hypertension and preeclampsia with OSA [52]. The study also showed that the frequency of sleep-disordered breathing was 1.4 times higher in women with gestational hypertension than controls without hypertension. The severity of sleep-disordered breathing among women with gestational hypertension or pre-eclampsia was more than twice as common compared to controls. These data should be helpful in identifying women at risk and controlling for the cardiovascular risk factors in women.

Moderate-to-severe obstructive sleep apnea syndrome is a common pathology in major depression. The identification of these different risk factors advances a new perspective for more effective screening of moderate to severe obstructive sleep apnea syndrome in major depression. Our study identified psychological comorbidities such as stress and depression, in addition to physical illnesses, in association with OSA. Indeed, in individuals with OSA, the prevalence of depressive affects may reach as high as 63% [55]. The co-existence of major depression and OSA may have a negative impact on quality of life. Further studies are needed to address the increasing morbidity of patients with coexistent OSA and depression.

In a large community-based prevalence study among 2400 adults in India [56], male gender, increasing age, obesity, and increased waist–hip ratio were identified as significant risk factors for OSA. Our study demonstrated the risk factors and prevalence for OSA in multiple countries, which are consistent with earlier findings. More importantly, we also identified a few modifiable risk factors for OSA, such as overweight/obesity, alcoholism, smoking, hypertension, diabetes mellitus, and hyperlipidemia. Given the fact that obesity is a major risk factor for OSA, and given the current global rise in obesity, the prevalence of OSA will increase in the future. More community-based educational interventions are needed for the reduction in the global epidemic of obesity.

### 4.1. Limitations

Due to the nature of the studies, no causal association could be made between OSA and the diseases. There were no clinical trials identified in this review. Although there were 10 prospective cohort studies reviewed, these studies did not report OSA or diseases as an outcome measurement. Future studies are needed to establish whether OSA can cause comorbidities and vice versa. Secondly, this systemic review included multiple structured searches using 10 sources. There is always a possibility that some of the important articles could have been missed. Again, the study did not aim to identify any relationships between specific traits of the population with OSA or any gender differences of OSA, although the study evaluated data obtained from multiple countries (e.g., European, Arab, Asian, American, Australasian) and in both genders. However, studies showed a higher severity of OSA in men [26]. Future studies are needed to establish relationships between OSA severity with traits and gender.

### 4.2. Strengths

This is a comprehensive systematic review comprising a large study population (*n* = 37,599) from 28 countries. The sources of bias in the literature selection were controlled using the PRISMA method of strict inclusion and exclusion criteria and involving an independent quality appraisal method using all three authors. The study results are generalizable to a wider population of multiple races and ethnicities.

## 5. Conclusions and Recommendations

This review provided substantial quantitative evidence to demonstrate the risk factors of OSA. Secondly, this study addressed an important public health problem of cardiovascular disease in association with OSA. The review also showed consistency of findings through multiple country-specific data and the strength of the association by providing evidence of rigorous quantitative analysis regarding the role of OSA in disease morbidity, both physically and psychologically. Reviewed studies consistently showed that severity of OSA, measured by the ACC/AHA risk calculator and by the AHI categories, determine the risk of several disease comorbidities including hypertension, gestational hypertension, diabetes, obesity, heart disease, stroke, asthma, and poor mental health. These results are in line with emerging evidence showing that patients with severe, untreated OSA have a higher incidence of cardiovascular events. Further studies are urgently needed to demonstrate specific behavioral, environmental, and biological mechanisms or pathways of disparities in OSA by race/ethnicity. Interventional studies are suggested based on the modifiable risk factors for OSA identified in this review.

## Figures and Tables

**Table 1:** Inclusion and Exclusion Criteria.

Inclusion Criteria	Exclusion Criteria
Human studies	Non-English articles
Scholarly articles	Studies that only involved ages 19 and younger
Age group: 19+	Review articles
Obstructive sleep apnea	Not human studies
Cardiovascular diseases	Studies that only involve other diseases besides hypertension and obstructive sleep apnea
Hypertension	Study period not between 2018–2021
Comorbidities; risk factors	

## Data Availability

Not applicable.
